# Hedgehog signaling regulates osteoblast differentiation in zebrafish larvae through modulation of autophagy

**DOI:** 10.1242/bio.040840

**Published:** 2019-04-16

**Authors:** Zhanying Hu, Bo Chen, Qiong Zhao

**Affiliations:** Institute of Medicinal Biotechnology, Chinese Academy of Medical Sciences and Peking Union Medical College, Beijing 100050, China

**Keywords:** Zebrafish, Autophagy, Hh signaling, Osteoblast, *atg5*, Mineralization, *Gli2*, *Ptch1*, *SHH*, BMP2

## Abstract

Impaired osteoblast differentiation may result in bone metabolic diseases such as osteoporosis. It was reported recently that hedgehog (Hh) signaling and autophagy are two important regulators of bone differentiation. In order to further dissect their relationship in bone development, we used a zebrafish larvae model to investigate how disruption of one of these signals affects the function of the other and impacts osteoblast differentiation. Our results showed that activation of Hh signaling negatively regulated autophagy. However, suppression of autophagy by knocking down *atg5* expression did not alter Hh signaling, but dramatically upregulated the expression of osteoblast-related genes and increased bone mineralization, especially in the den region. On the contrary, inhibition of the Hh signaling pathway by cyclopamine treatment suppressed the expression of osteoblast-related genes and decreased bone mineralization. In agreement with these findings, blocking Hh signaling through knockdown *SHH* and *Gli2* genes led to defective osteoblast differentiation, while promoting Hh signaling by knockdown *Ptch1* was beneficial to osteoblast differentiation. Our results thus support that activation of the Hh signaling pathway negatively regulates autophagy and consequentially promotes osteoblast differentiation. On the contrary, induction of autophagy inhibits osteoblast differentiation. Our work reveals the mechanism underlying Hh signaling pathway regulation of bone development.

## Abstract (precis)

**Summary:** Our report of an essential regulation role of hedgehog signaling and autophagy on osteoblast differentiation may contribute to research on bone development biology, hedgehog signaling and the autophagy pathway.

## INTRODUCTION

Osteoblasts are responsible for bone formation, and impaired osteoblast development leads to serious bone diseases such as osteoporosis (Edsall and Franz-Odendaal, 2010). Zinc finger-containing transcription factor Osterix (Sp7) is the main regulator of osteoblastgenesis. Loss of Sp7 leads to delay of bone maturation and dysregulates bone formation (Li et al., 2009; Nakashima et al., 2002; Kague et al., 2016). Bone morphogenetic protein 2 (BMP2) is another positive regulator, which stimulates osteoblast differentiation, maturation and osteoblast growth (Zhao et al., 2017; Kuo et al., 2006).

Autophagy is an intracellular degradation system, and is responsible for degrading and recycling aggregated proteins, damaged organelles, misfolded proteins and long-lived proteins (Hocking et al., 2012; Mizushima, 2007). During osteoblast differentiation, rapid synthesis of bone-matrix protein results in accumulation of misfolded proteins, and high autophagy activity is thus required for their removal (Hocking et al., 2012). Autophagy also plays a regulatory role in osteoblast formation and differentiation, although both inhibitory and promoting effects on osteoblast differentiation were observed in different experimental systems. For instance, pneumolysin-induced autophagy inhibited osteoblast differentiation, and treatment with autophagy inhibitors or knockdown of *atg5* alleviated the PLY-induced inhibition of differentiation (Kim et al., 2017). Stimulation of autophagy promoted osteoblast differentiation, and suppression of autophagy inhibited osteoblast terminal differentiation in mice (Ha et al., 2014; Liu et al., 2013). Moreover, inhibition of autophagy by depletion of Atg7 in the osteoblast lineage led to low bone mass and fractures associated with reduced numbers of osteoblasts (Piemontese et al., 2016). Promoting autophagy level by the mTOR pathway inhibited osteoblast apoptosis (Yang et al., 2013).

The hedgehog (Hh) signaling pathways, mediated by sonic hedgehog (Shh) and Indian hedgehog (Ihh), are recognized as indispensable regulators for osteoblast differentiation and morphological transition. Specifically, the transmembrane receptor Patched (Ptch1) binding to a secreted ligand initiates Hh signaling, whereas unliganded Ptch1, as a negative regulator of Hh signaling, inhibits the activity of the membrane protein Smoothened (Smo), which regulates downstream Gli transcriptional effectors. Among three Gli proteins, Gli2 is a very important activator for the Hh signaling pathway (Hui and Angers, 2011; Cohen et al., 2015; Sasaki et al., 1997). Activation of both Hh signaling pathways could positively regulate osteoblast differentiation. Shh promotes interactions between epidermal cells and osteoblast progenitors, which affect the shape of regenerated zebrafish bone. Ihh regulates the function of bone morphogenetic protein and further affects chondrocyte and osteoblast differentiation through Gli2 transcription factors. Ihh signaling modulates bone shaping during early morphogenesis of zebrafish craniofacial skeleton (Armstrong et al., 2017; Cai and Liu, 2016; Hojo et al., 2013; Huycke et al., 2012; Marumoto et al., 2017). Although mature osteoblasts were still in long bones of the Ihh deficient mutant mice limbs, growth plate formation was completely lost (Amano et al., 2015). Purmorphamine and other new Hh agonists triggered the Hh signaling pathway in hMSCs, resulting in an increase of osteoblast differentiation (Oliveira et al., 2012; Nakamura et al., 2015). Cyclopamine (cyA), an inhibitor of Hh signaling, decreased bone mass in adult mice (Ohba et al., 2008). However, another study showed that constitutive activation of Hh signaling impaired bone formation in mice (Joeng and Long, 2013).

Interestingly, accumulating evidence suggests that the Hh signaling pathway cross-talks with autophagy pathway. For example, Hh signaling inhibited formation of autophagosome both in basal level and in autophagy-induced conditions (Jimenez-Sanchez et al., 2012). In pancreatic cancer cells and breast cancer cells, activation of Hh signaling inhibits autophagy, while inhibition of Hh signaling promotes autophagy (Xu et al., 2014; Wang et al., 2017). In the Shh intestinal epithelial conditional knockout mouse model, autophagic levels decreased significantly in hippocampal neurons and vascular smooth muscle cells, indicating that the Shh signaling pathway may be involved in autophagy (Gagné-Sansfaçon et al., 2014; Petralia et al., 2013; Li et al., 2012). These findings inspired us to speculate that the Hh signaling and authophagy pathways, two important regulatory pathways of osteoblast development, may coordinate to regulate osteoblast differentiation. We therefore set out to investigate the interaction between Hh signaling and autophagy pathways and their impacts on bone development in zebrafish larvae.

As an animal model of bone development, *Danio rerio* (zebrafish) has a high similarity with human in bone architecture, bone cell types (osteoblasts and osteoclasts) and matrix proteins (Pasqualetti et al., 2012). Type X collagen (col10a1), a molecular marker of bone tissue, is expressed in both endochondral and intramembranous bones of zebrafish. Kim and colleagues established a col10a1:GFP transgenic zebrafish line that specifically expresses GFP in osteoblasts. This transgenic zebrafish is a useful tool for investigating osteoblast formation and differentiation (Avaron et al., 2006; Kim et al., 2013). In recent years, several reports have shown that Hh signaling promotes osteoblast differentiation in the zebrafish regeneration model (Armstrong et al., 2017; Paul et al., 2016; Blum and Begemann, 2015), however, how the Hh signaling pathway regulates the osteoblasts’ differentiation in zebrafish larvae remains to be investigated. Using the zebrafish model, we demonstrate that the Hh signaling pathway suppresses autophagy, whereas the autophagy pathway didn't affect Hh signaling. Furthermore, we found that whereas autophagy inhibits osteoblast differentiation, activation of the Hh signaling pathway promotes osteoblast differentiation and development. Our results thus favor a hypothesis that suppression of osteoblast differentiation by the Hh signaling pathway inhibits autophagy, which consequentially promotes osteoblast differentiation.

## RESULTS

### Hh pathway suppressed autophagy level in zebrafish larvae

#### Autophagy had no obvious effect on Hh signaling

**Fig. 1.**
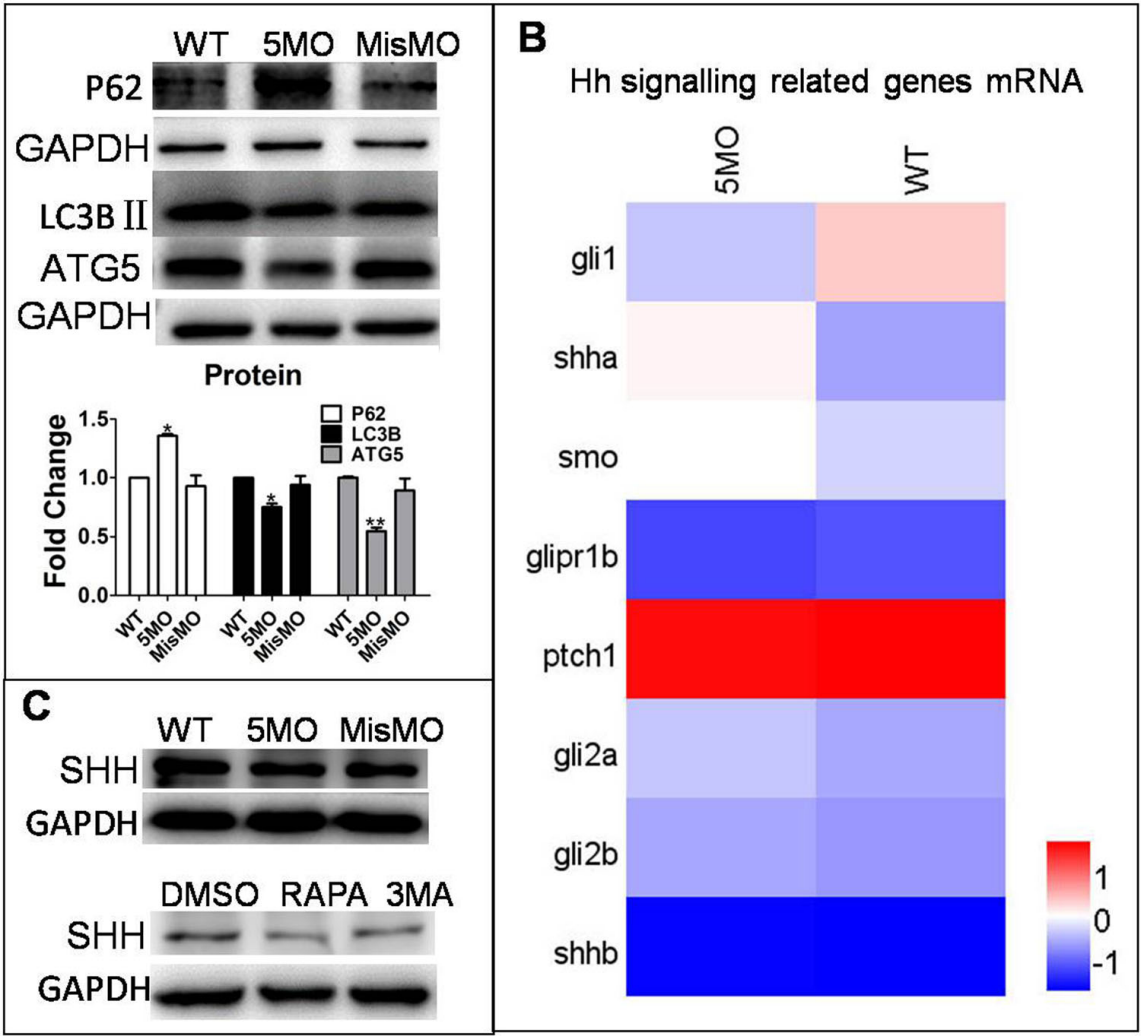
**Autophagy did not regulate the Hh signaling pathway.** ATG5MO and MisMO were injected into embryos at the 1–4 cell stage. MisMO-injected group as a negative control. The means and s.d. were derived from triple duplicates. (A) Expression of LC3BII and ATG5 proteins was determined by a western blot assay. GAPDH served as a loading control. (B) Heat map of mRNA expression of Hh signaling pathway-related genes *Smo*, *Gli* family number *Gli1*, *Gli2a*, *Gli2b*, *ptch1* and *Shh* mRNA from wild-type and ATG5MO-injected embryos was determined by RNAseq assay. (C) Embryos at 1–4 cell stage were either injected with ATG5MO or MisMO or mock-treated and treated with 10 µM of RAPA or 2 mM of 3MA for 3 days from 3 dpf. Expression of SHH protein was determined by a western blot assay. GAPDH served as a loading control.

To investigate the relationship between autophagy and the Hh signaling pathway, autophagy-related gene *atg5* was knocked down by morpholino oligos, which resulted in down- and upregulation of autophagy marker proteins LC3BII and P62, respectively (Fig. 1A). However, *atg5* knockdown did not apparently alter the transcriptional level of Hh signaling pathway-related Shh genes, such as membrane receptors Smo, Ptch1 and downstream transcriptional effectors Gli2, although *shha* was induced by *atg5* knockdown, but *shhb* had no change, and *gli1* was decreased by 5MO, but *gli2a* and *gli2b* had no change (Fig. 1B). Furthermore, neither injection of ATG5MO, treatment with autophagy inducer rapamycin (RAPA) nor inhibitor 3MA changed the level of SHH protein expression (Fig. 1C). These results collectively suggest that autophagy has no obvious effect on the Hh signaling pathway.

#### Hh signaling pathway negatively regulated autophagy activity

**Fig. 2.**
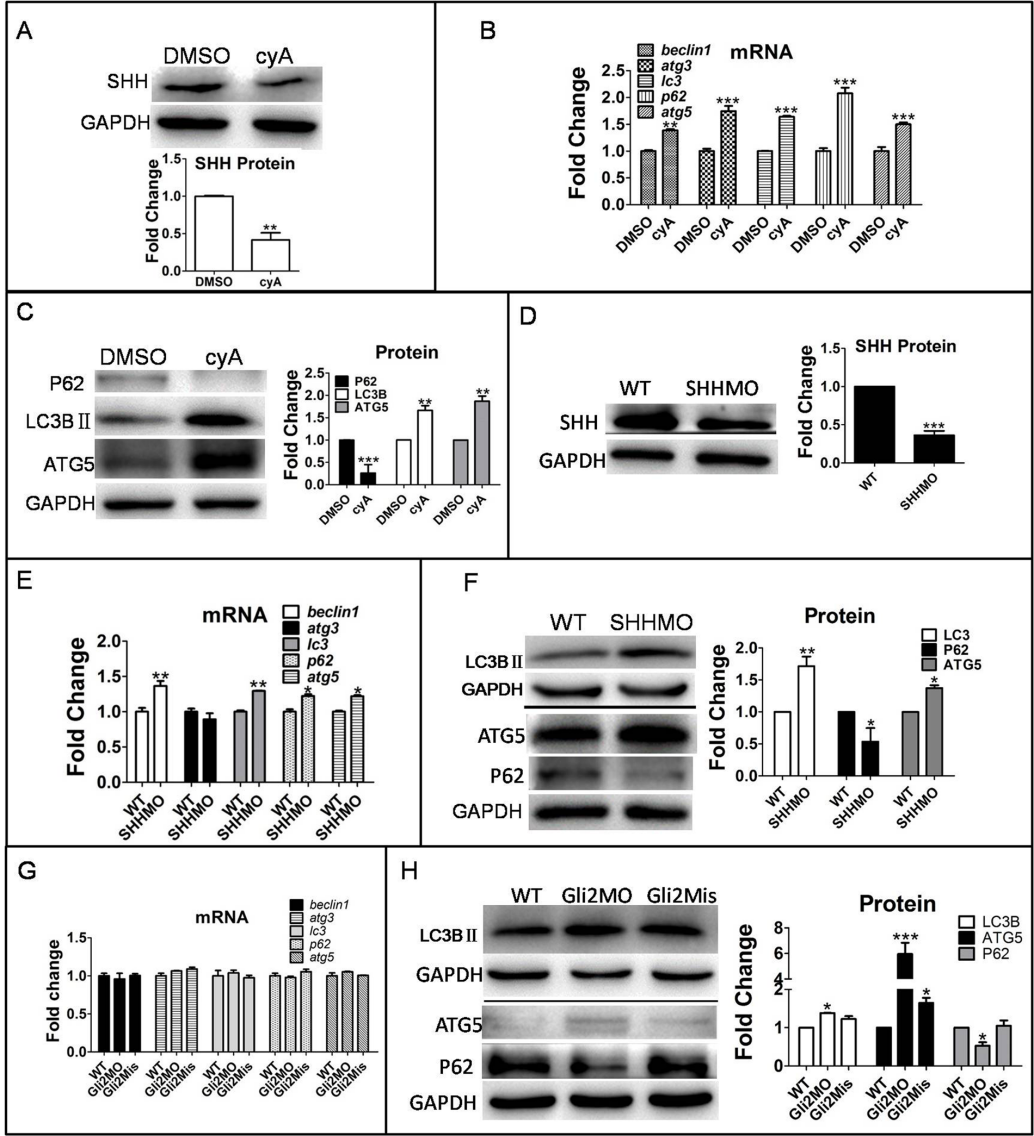
**Autophagy level was promoted by downregulation of the Hh signaling pathway in zebrafish larvae.** (A–C) Larvae were exposed to 10 µM cyA and 0.1% DMSO at 3 dpf for 3 days. (D–H) Embryos were injected with SHHMO, Gli2MO and Gli2Mis at the 1–4 cell stage; at 6 dpf, larvae were collected for assays . GAPDH served as a loading control. The means and s.d. were derived from triple duplicates. *P<0.05, **P<0.01, ***P<0.001 versus the WT groups or DMSO control groups (one-way ANOVA). The data were from three independent experiments. (A,D) SHH protein was detected by western blot assay. (B,E,G) Transcriptional level of autophagy-related genes was tested by qPCR assays. (C,F,H) Autophagy-related proteins LC3B II, ATG5 and P62 level were determined by western blot.

In order to investigate the effects of the Hh signaling pathway on autophagy in zebrafish, we disrupted the Hh signaling pathway via three different approaches. First, treatment of zebrafish larvae with cyclopamine (cyA), an Hh signaling pathway inhibitor, for 3 days at 3 dpf downregulated SHH protein expression (Fig. 2A), but expression of autophagy-related genes, including *beclin1*, *atg3*, *lc3*, *p62* and *atg5*, were upregulated at both mRNA and protein levels (Fig. 2B). Furthermore, LC3BII and ATG5 proteins were upregulated, whereas p62 protein was downregulated by cyA treatment (Fig. 2C). Second, the Hh signaling pathway was downregulated by suppressing the expression of *shh* and *gli2*, Hh signaling positively regulated genes by injection of Shh morpholino oligonucleotide (MO) and Gli2MO, respectively. Gli2Mis was used as a negative control. Consistent with results observed under cyA treatment condition, downregulation of SHH and gli2 expression (Fig. 2D) increased the transcripts of autophagy-related genes *beclin1*, *lc3*, *p62* and *atg5* (Fig. 2E) and autophagy-related protein LC3BII and ATG5, but decreased the level of p62 protein (Fig. 2F–H). Those two experiments strongly suggest that inhibition of the Hh signal pathway increases autophagy activity.

**Fig. 3.**
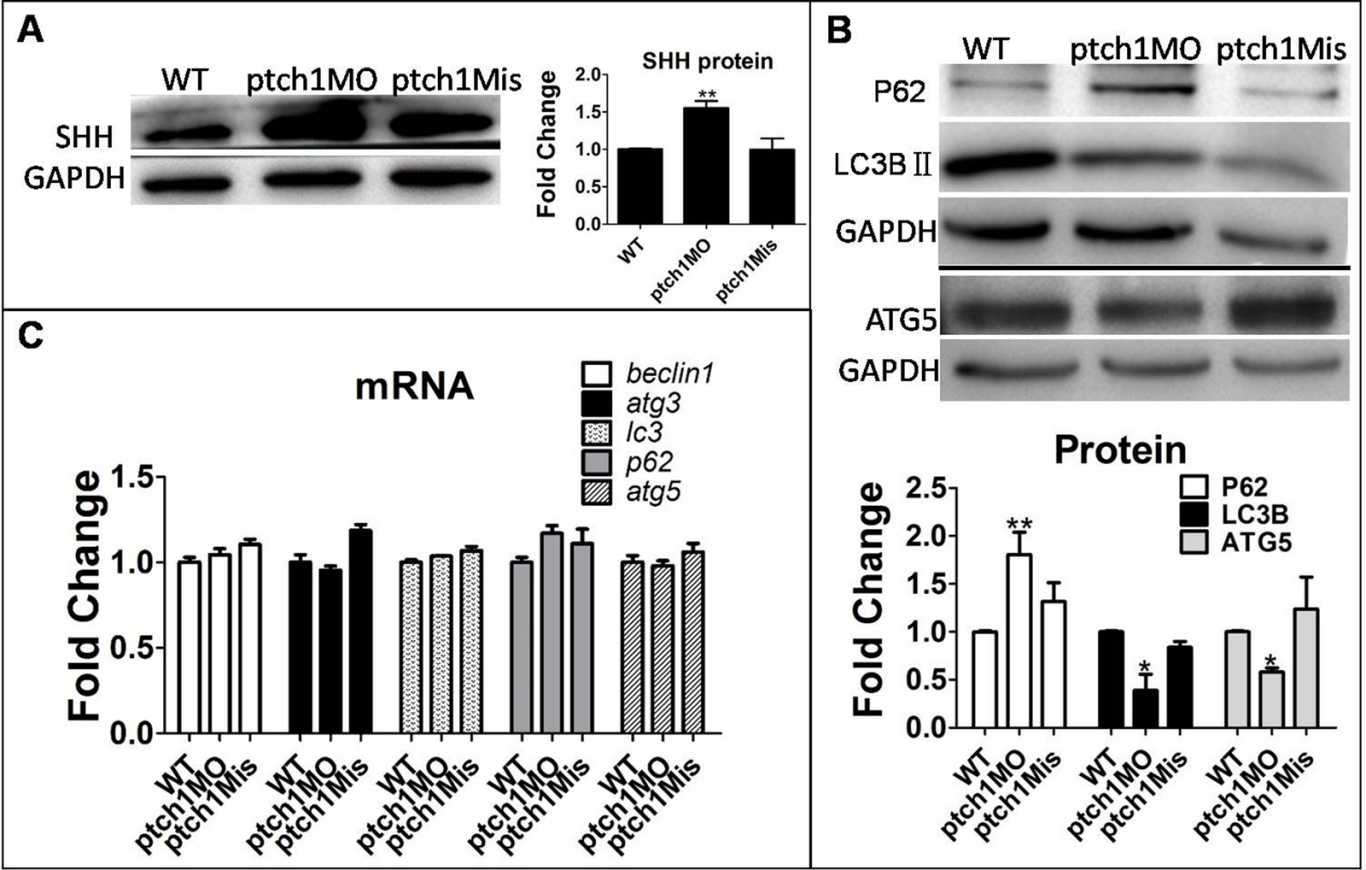
**Promotion of Hh signaling by downregulated *Ptch1* gene suppressed autophagy level.** Embryos were injected with ptch1MO and ptch1Mis at the 1–4 cell stage and larvae were collected at 6 dpf for qPCR and western blot. GAPDH as a loading control. The means and s.d. were derived from triple duplicates. (A,B) SHH protein and autophagy-related proteins were detected by western blot. (C) Ptch1MO-injected group compared with WT group and ptch1Mis-injected group. Autophagy gene mRNA levels were tested by qPCR. *P<0.05, **P<0.01 versus WT groups (one-way ANOVA).

Third, to further confirm the regulatory effect of the Hh signaling pathway on autophagy, we activated the Hh signaling pathway by knocking down *Ptch1*, the Hh signaling negative regulator, by injection of Ptch1MO or Ptch1Mis into 1–4 cell stage embryos. As anticipated, knockdown of *Ptch1* expression increased SHH protein and autophagy protein p62, but decreased the autophagy proteins ATG5 and LC3BII (Fig. 3).

Taken together, the results presented in this section consistently support the notion that Hh signaling negatively regulates autophagy and may further inhibit autophagy level.

### Autophagy negatively regulated osteoblast differentiation in zebrafish

**Fig. 4.**
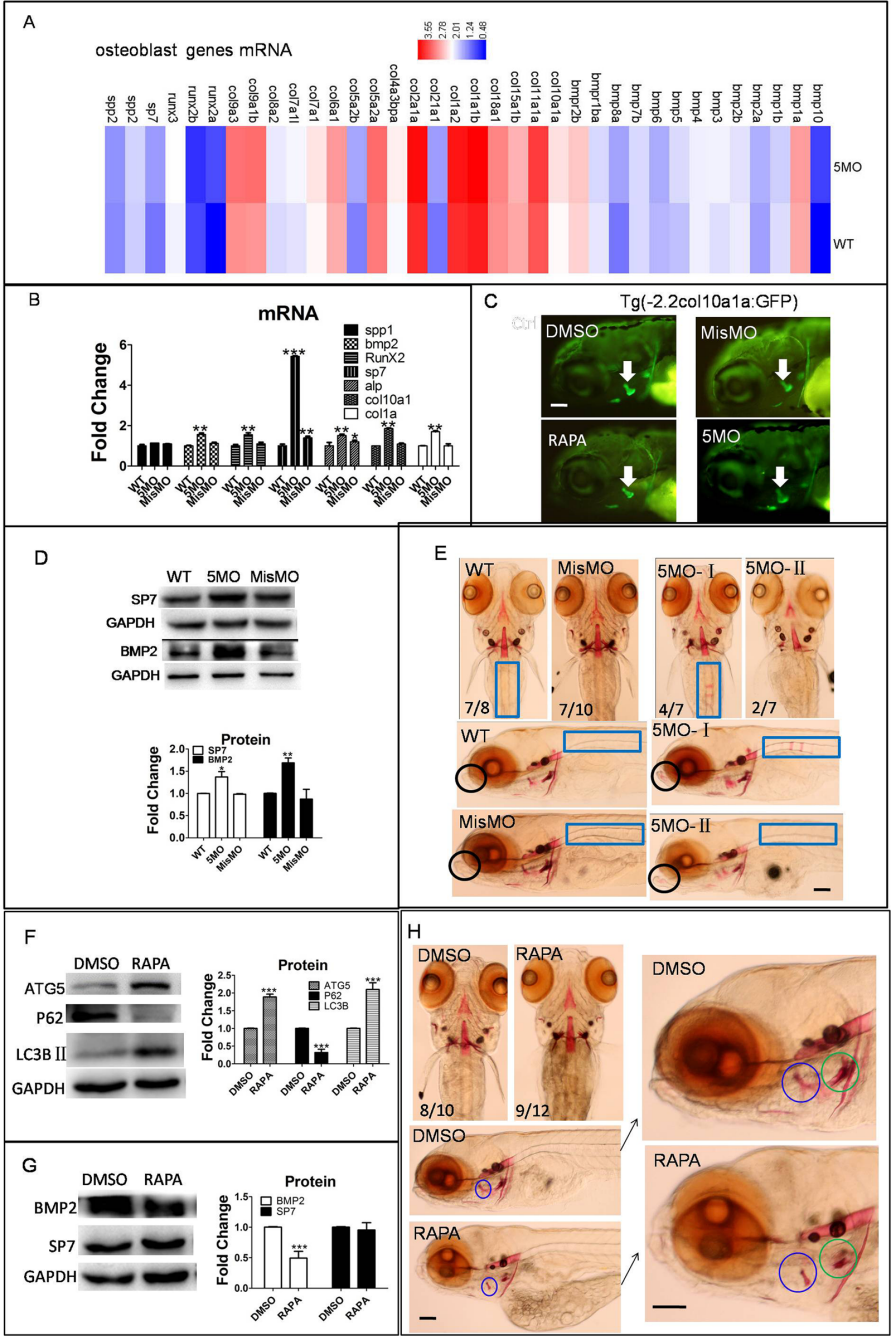
**Osteoblast differentiation was regulated by autophagy in zebrafish larvae.** (A–E) Embryos or transgenic Tg (-2.2col10a1a:GFP) embryos were injected with 5MO and MisMO at the 1–4 cell stage, and larvae were collected at 6 dpf for transcriptom sequencing, qPCR, western blot and Alizarin Red staining, transgenic larvae were collected at 4 dpf for image capture. (A) Heat map showed osteoblast-related genes mRNA level, data were from transcriptome sequencing. (B) Transcriptional level of osteoblast genes were detected by qPCR. (C) Col10a1a expression pattern in transgenic zebrafish larvae at 4 dpf. (D) Osteoblast proteins SP7 and BMP2 were detected by western blot. (E) Alizarin Red staining showed the mineralization of osteoblast at larvae den and rib. 5MO-II:abnormal phenotype. (F–H) Larvae were exposed to 50 µM RAPA at 3 dpf, larvae were collected at 6 dpf for western blot and staining assay separately. (F,G) Western blot assay showed autophagy proteins and osteoblast proteins were changed by RAPA. (H) Alizarin Red staining showed osteoblast mineralization. Black circle, dentary (den); blue rectangle, rib; blue circle, opercle (op); green circle, ceratobranchial 5 (cb5). The means and s.d. were derived from triple duplicates, *P<0.05, **P<0.01, ***P<0.001 versus WT groups (one-way ANOVA). The data of western blots were from three independent experiments. Scale bars: 50 µm.

To understand the role of autophagy in osteoblast differentiation, autophagy level was blocked by knockdown autophagy gene *atg5*. Results showed osteoblast genes *runx2a*, *bmp2*, *bmp8*, *bmp10*, *sp7*, *col1a*, *alp* and *col10a1a* were upregulated at transcription level by 5MO. We also showed the regulation of atg5 to many other bmp family genes and col family genes (see details from GEO database, Fig. 4A,B). BMP2 and SP7 protein were also dramatically upregulated by 5MO (Fig. 4D), the protein expression of col10a1a in the op region were increased during the inhibition of autophagy at 4 dpf zebrafish larvae (Fig. 4C). Furthermore, Alizarin Red staining assay demonstrated that osteoblast mineralization of the rib and dentary regions was increased by *atg5* knockdown at 6 dpf zebrafish larvae, the den region mineralization was observed even in abnormal phenotype, although the total staining was reduced in 5MO- abnormal group (Fig. 4E).

Next, RAPA, an inducer of autophagy, was used to treat zebrafish larvae. An increase of autophagy on osteoblasts was observed with upregulation of ATG5 and downregulation of P62 (Fig. 4F). However, col10a1a protein in the op region was reduced by RAPA treatment. Western blot results showed that BMP2 protein was downregulated, but SP7 protein had no obvious change (Fig. 4G). The osteoblast mineralization was reduced by RAPA, especially at the op region (Fig. 4H).

In summary, our results demonstrate that inhibition of autophagy induced osteoblast-related gene upregulation in transcription and protein levels, and promoted osteoblast mineralization. On the contrary, increasing autophagy by RAPA inhibited osteoblast gene expression and reduced osteoblast mineralization. These results imply that autophagy negatively regulates osteoblast differentiation.

### Activation of the Hh signaling pathway promoted osteoblast differentiation in zebrafish larvae

#### Hh signaling pathway inhibitor cyA suppressed osteoblast differentiation in zebrafish larvae

**Fig. 5.**
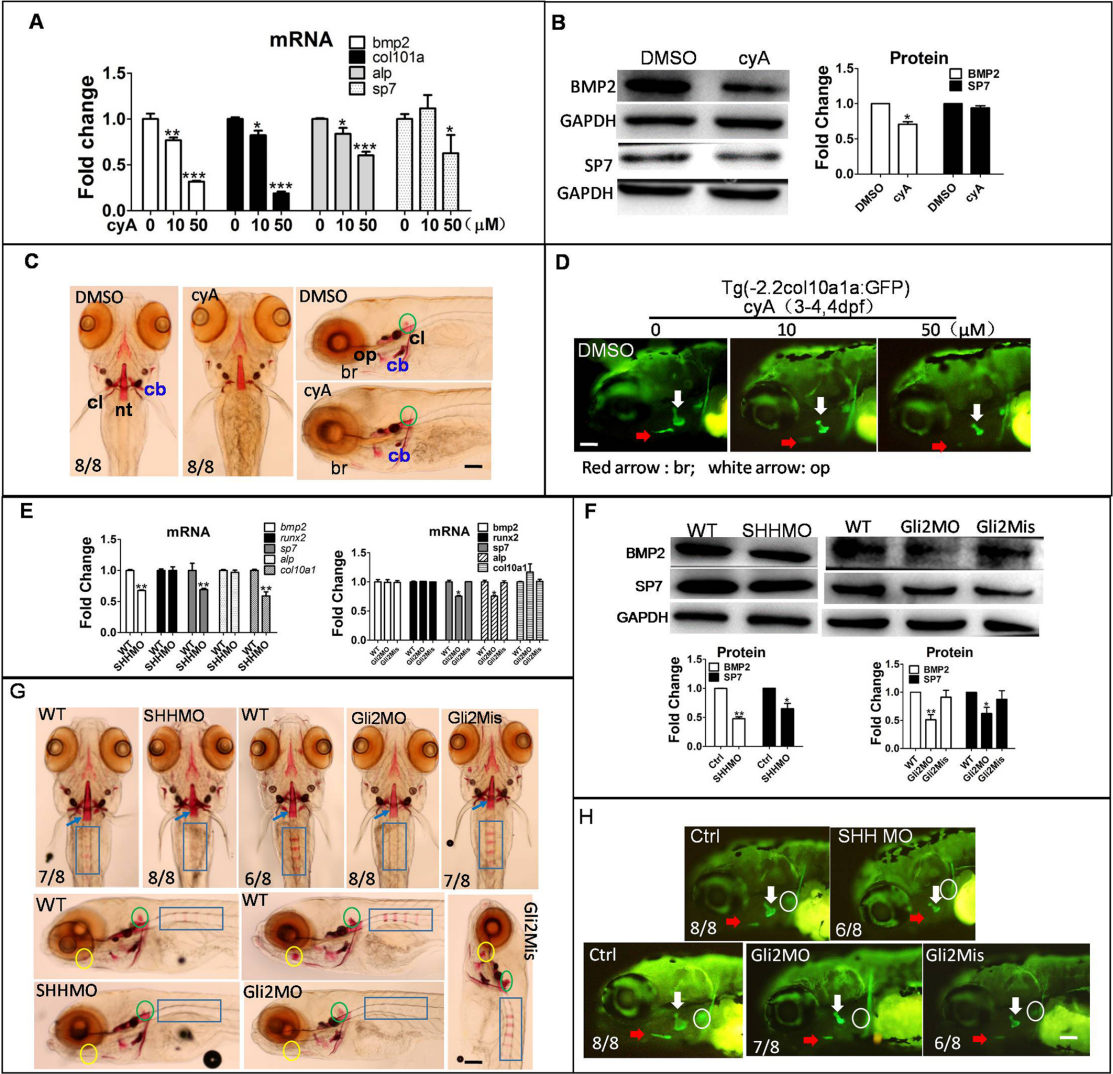
**Reduction of Hh signaling inhibited osteoblast differentiation.** Larvae were exposed to 10 µM and 50 µM cyA at 3 dpf for 3 days, and were collected to measure osteoblast-related gene mRNA, protein level and mineralization of osteoblasts with DMSO treated as a control. (A) qPCR detected osteoblast genes in transcription level. (B) BMP2 and SP7 proteins were detected by western blot. (C) Alizarin Red staining showed mineralization of osteoblasts. Note that the DMSO panel on the right is reproduced from middle WT panel in Fig. 4E. (D) Transgene larvae Tg (-2.2col10a1a:GFP) were treated with 10 µM and 50 µM cyA at 3 dpf for 1 day, images were obtained by fluorescence microscope at 4 dpf. Embryos were injected with SHHMO, Gli2MO and Gli2Mis at the 1–4 cell stage. Lateral view. (E–H) At 6 dpf, larvae were collected for qPCR, western blot and Alizarin Red staining. (E) Osteoblast genes *bmp2*, *sp7*, *col10a1*, *runx2* and *alp* mRNA were detected by qPCR. (F) Osteoblast-related proteins BMP2 and SP7 were tested by western blot. (G) Alizarin Red staining showed mineralization of osteoblasts including nt (notochord tip, blue arrow), rib (blue rectangle), hm (hyomandibular, yellow circle) and regions delayed in SHHMO- and Gli2MO-injected groups. Green circle, bop (basioccipital articulatory process). The difference in osteoblast name and abbreviation were derived from previous sources (Laue et al., 2008; Aceto et al., 2015). (H) In Tg (-2.2col10a1a:GFP) transgene zebrafish, col10a1a signal was shown. White circle, cb (ceratobranchial 5); red arrow, br; white arrow, op. *P<0.05, **P<0.01, ***P<0.001 versus untreated groups (one-way ANOVA). The data were from three independent assays. Scale bars: 50 µm.

To investigate the effect of Hh signaling inhibitor cyA on the osteoblast differentiation, zebrafish larvae were exposed to cyA at 3 dpf. qPCR results showed osteoblast-related genes *bmp2*, *col10a*, *alp* and *sp7* mRNA were downregulated in a dose-dependent manner (Fig. 5A). col10a1a: GFP transgenic zebrafish line was used to detect col10a1a protein location and expression and, after cyA treatment, col10a1a protein was inhibited in the br and op regions in a dose-independent manner (Fig. 5D) and BMP2 and SP7 protein were downregulated by cyA (Fig. 5B). Alizarin Red staining showed mineralization of osteoblasts was obviously suppressed by cyA (Fig. 5C), particularly in the cb region as well as in the cl, nt and op regions, indicating that osteoblast differentiation was decreased, which is consistent with the results in Fig. 5D.

#### Osteoblastic differentiation was reduced after knocking down *SHH* and *Gli2* genes in zebrafish larvae

To examine the function of the Hh signaling pathway in osteoblasts, we chose to decrease Hh signaling by injecting SHHMO and Gli2MO into zebrafish embryos at the 1–4 cell stage. In SHHMO-injected groups, results showed that BMP2, SP7 and col10a1a were downregulated in mRNA and protein levels (Fig. 5E,F,H), Furthermore, knockdown of *SHH* reduced col10a1a protein in the op and br regions (Fig. 5H). Positive positions in rib and bop were reduced by SHHMO (Fig. 5G). Transcriptional factor Gli2 in Hh signaling downstream did not affect osteoblast-related gene mRNA, but downregulated BMP2 and SP7 protein abundance (Fig. 5E,F). Col10a1a in the op, cl and cb regions were reduced by Gli2MO (Fig. 5H), and in Gli2MO-injected groups, Alizarin Red staining at rib and hm were decreased, indicating that Gli2MO inhibited osteoblast mineralization.

The above results suggest that osteoblast mineralization is blocked or decreased after suppressing Hh signaling by compound cyA or by knockdown *Shh* and *Gli2* genes.

#### Upregulation of Hh signaling by ptch1 MO-induced osteoblast differentiation

**Fig. 6.**
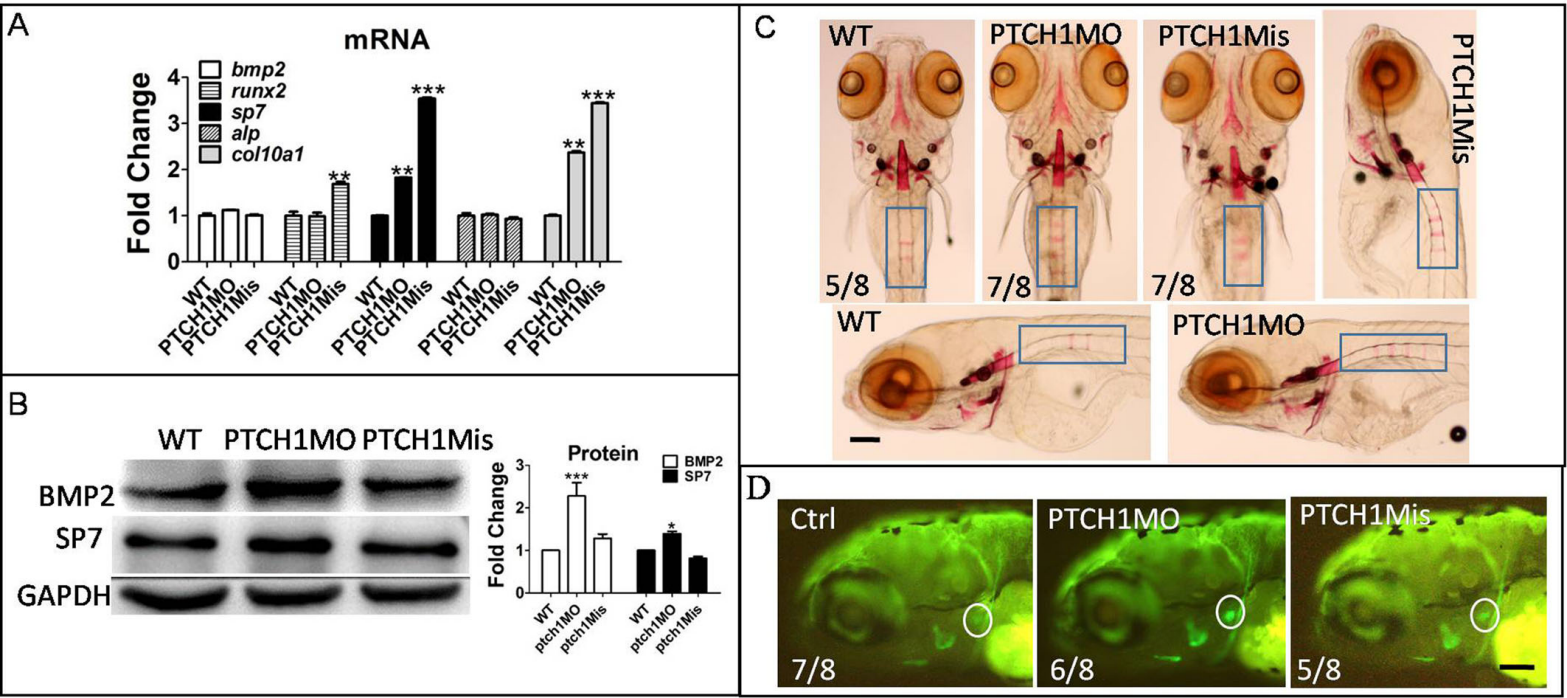
**Increasing Hh signaling by Ptch1 knockdown might be beneficial for osteoblast development.** Embryos were injected with Ptch1MO and Ptch1Mis at the 1–4 cell stage, at 6 dpf, larvae were collected for qPCR, western blot and Alizarin Red staining respectively. (A) QPCR showed osteoblast genes mRNA level. (B) Western blot results showed BMP2 and SP7 protein level. (C) Alizarin Red staining showed bone mineralization; blue rectangle, rib. (D) Col10a1a protein expression in cb5 (ceratobranchial 5, white circle) region was observed in Ptch1MO-injected group. *P<0.05, **P<0.01, ***P<0.001 versus the untreated groups (one-way ANOVA). The data were from three independent assays.

To further explore the role of Hh signaling in osteoblast differentiation, we activated Hh signaling by knockdown *ptch1*. Results showed *sp7* and *col10a1* mRNAs were upregulated in ptch1MO-injected groups, but *bmp2*, *runx2* and *alp* mRNA did not change (Fig. 6A). BMP2 and SP7 proteins increased in the ptch1MO-injected group (Fig. 6B). The col10a1 protein located in the cb region was increasing in the ptch1MO-injected group (Fig. 6D). The mineralization of larvae rib was promoted in ptch1 group compared to wild-type (WT) and ptch1Mis groups (Fig. 6C).

### Activation of autophagy and inhibition of Hh signaling synergistically impair bone development in zebrafish larvae

**Fig. 7.**
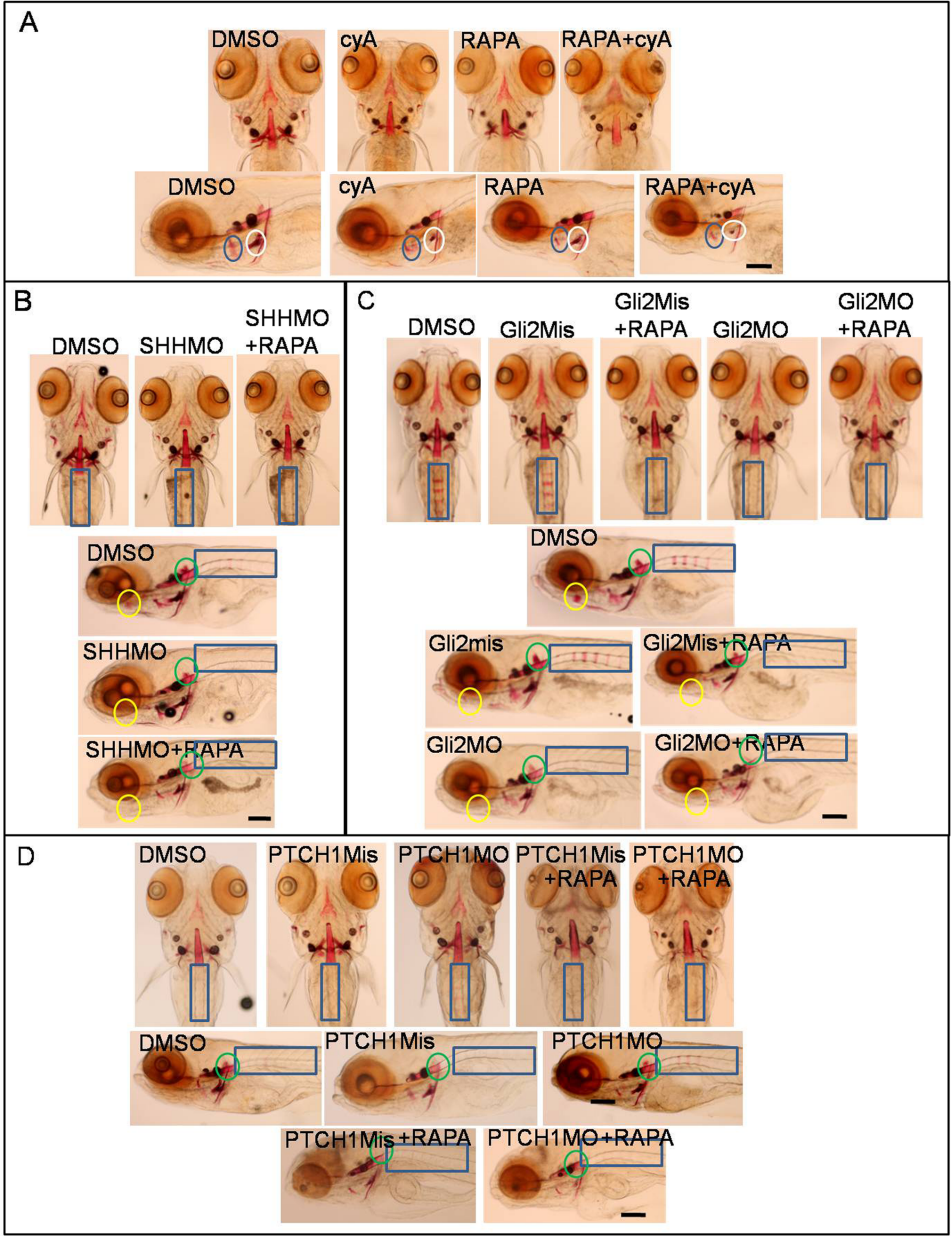
**Autophagy inducer RAPA and block of Hh signaling co-inhibited osteoblast mineralization.** (A) Larvae at 3 dpf were exposed to 10 µM cyA, 10 µM RAPA or cyA plus RAPA, with larvae collected for Alizarin Red staining at 6 dpf. Blue circle, op; white circle, cb. (B–D) Embryos were injected with 50 µM SHHMO, Gli2MO, Gli2Mis, PTCH1MO and PTCH1Mis at the 1–4 cell stage, DMSO control group and injected group larvae were treated with 10 µM RAPA, with larvae collected at 6 dpf for Alizarin Red staining. Yellow circle, bop; green circle, hm; blue rectangle, rib. Scale bars: 50 µm.

To further reveal the role of autophagy level and Hh signaling in osteoblast mineralization, larvae at 3 dpf were treated by cyA, RAPA and cyA plus RAPA for 3 days, staining by Alizarin Red showed cyA mainly inhibited cb5 region osteoblast mineralization, RAPA suppressed osteoblast mineralization in the op area. However, combination treatment of RAPA and cyA inhibited osteoblast mineralization in broad areas, including the op, cb5, nt, hm regions and so on (Fig. 7A). Moreover, with suppression of Hh signaling by knockdown of *SHH* and *Gli2* genes, at 3 dpf, larvae were exposed to RAPA for 3 days, and then were collected for Alizarin Red staining. Results showed osteoblast mineralization of the rib and hm regions is less in the SHHMO-injected group than in DMSO control, however RAPA-treated SHHMO-injected group intensified osteoblast mineralization loss in the rib and hm regions compared to SHHMO-injected group. Osteoblast staining reduced in rib, hm and bop regions in RAPA plus Gli2MO group compared with WT, Gli2MO, Gli2Mis and Gli2mis plus RAPA groups (Fig. 7B,C). While promotion of Hh signal by knockdown *PTCH1* enhanced osteoblast mineralization, it was blocked by autophagy inducer RAPA (Fig. 7D). In this section, data suggest that the inhibition of the Hh signaling pathway and activation of autophagy synergistically suppressed bone development and osteoblast mineralization. However, the bone mineralization enhancement, dependent on Hh signal activation, was blocked by activation of autophagy.

## DISCUSSION

**Fig. 8.**
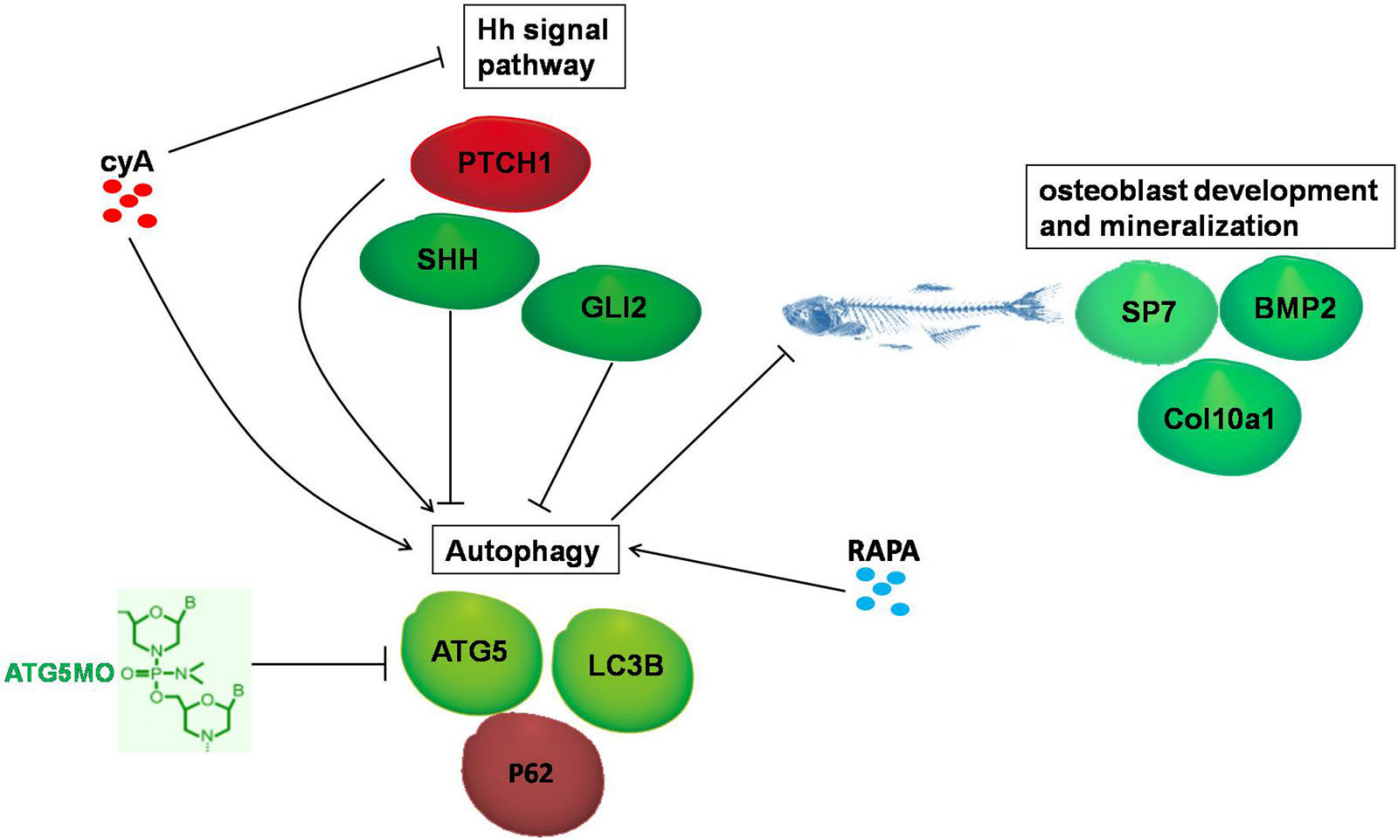
**Schematic illustrates the interaction between Hh signaling and autophagy, and their regulated roles on osteoblast differentiation and mineralization.**

Reports about the relationship between Hh signaling and autophagy exist paradoxically in cell models. For instance, in hippocampal neurons and intestinal secretory cells, reports indicate the Hh signaling pathway promotes autophagy (Petralia et al., 2013; Gagné-Sansfaçon et al., 2014), but studies in human hepatocellular carcinoma cells, pancreatic ductal adenocarcinoma cells and cancer cells suggest that inhibition of Hh signaling induces autophagy and that autophagy is a key factor for the Hh signaling pathway to regulate a variety of biological functions (Wang et al., 2013; Xu et al., 2014; Li et al., 2012; Tang et al., 2015; Jimenez-Sanchez et al., 2012). It is probable that Hh signaling has completely different roles on autophagy depending on cell types and patho-physiological conditions. In this study, we dissected the interaction between Hh signaling and autophagy *in vivo* zebrafish model. Our results clearly indicate that autophagy has no influence on Hh signaling, whereas Hh signaling negatively regulates autophagy (Fig. 1). Specifically, inhibition of Hh signaling by pharmacological and knockdown approaches consistently promoted autophagy activity, while activation of Hh signaling by knocking down the expression of its negative regulator Ptch1 protein inhibited autophagy. Interestingly, inhibition or activation of Hh signaling was associated with up- or downregulation of autophagy-related protein ATG5, respectively (Figs 2 and 8). Our results suggest that the Hh signaling pathway is an upstream regulator of autophagy, at least in zebrafish osteoblasts. This notion is in agreement with an early report that the autophagy upstream inducer beclin1 was regulated by the Hh signaling pathway through Gli2 expression (Won et al., 2015). In this study, *Gli2* knockdown could indeed increase autophagy proteins ATG5 and LC3B. Whether BECLIN1 mediates the Hh signaling pathway regulation of autophagy in zebrafish remains to be determined.

Autophagy was reported to play an essential role in bone development. For instance, deleting ATG7, an essential factor in autophagy level, in mice led to low bone mass and reduction of osteoblast number (Piemontese et al., 2016; Bartolomé et al., 2013). However, in agreement with our findings reported herein, other studies showed that autophagy played a negatively regulatory role in osteoblast differentiation. Specifically, suppression of autophagy by inhibitors or autophagy-related gene knockdown could increase osteoblast differentiation and activity (Kim et al., 2017; Whitehouse et al., 2010). On the other hand, autophagy inducer RAPA dramatically inhibited osteoblast differentiation and mineralization (Singha et al., 2008; Isomoto et al., 2007). In this study, we obtained several lines of evidence suggesting that autophagy does regulate osteoblast differentiation in zebrafish larvae. Knockdown autophagy gene *atg5* promoted osteoblast genes and proteins expression, and increased osteoblast mineralization. Also, autophagy inducer RAPA treatment led to BMP2 reduction, while osteoblast mineralization in the op and cb regions were decreased, especially in the op region of zebrafish larvae (Fig. 4).

RAPA is an mTORC1 inhibitor, mTORC1 had been demonstrated to be an important kinase for bone formation (Sugamori et al., 2016). As an autophagy inducer, RAPA also promotes ATG5 and LC3B expression (Hu et al., 2011) and our data indicate as such. Also we report that RAPA inhibits P62 protein level in zebrafish larvae (Fig. 4). It is thus possible that RAPA suppresses osteobalstic differentiation and mineralization through both the mTORC1 and autophagy pathways.

Hh signaling plays a vital role in osteoblast differentiation and mineralization (Bae et al., 2016; Nakamura et al., 2015). Shh has the ability to induce ectopic cartilage and bone formation *in vivo* (Iwamoto et al., 1999) and stimulates osteoblastic differentiation mainly through upregulation of *SP7* gene expression in osteoblastic cells (Tian et al., 2012), Hh signaling-related zinc finger transcription factor Gli2 mediates BMP2 expression in osteoblasts (Hojo et al., 2013; Zhao et al., 2006). Our data supports that Hh signaling promotes osteoblastic differentiation and mineralization in the zebrafish larvae model. Firstly, SHH dysfunction downregulated BMP2, SP7 and col10a1 expression, in addition osteoblastic mineralization located in rib, hm, op and cb5 regions of zebrafish larvae were damaged. Secondly, Gli2 dysfunction resulted in downregulation of BMP2 and SP7 protein, and reduction of bone mineralization area. The conclusion was identified in a zebrafish fin regeneration study by Armstrong et al. (2017). Thirdly, Hh signaling inhibitor cyA downregulated osteoblast gene BMP2, SP7 and col10a1 in transcription and translation levels – following osteoblastic mineralization – decreased, especially the mineralization located in the cb5 region, which was strongly reduced in zebrafish larvae. Fourthly, promoting Hh signaling by knockdown *ptch1* gene, resulted in upregulation of osteoblast-related proteins.

In summary, using the zebrafish larvae model, we illustrated that the autophagy pathway has no obvious role in regulating the Hh signaling pathway, but the Hh signaling pathway negatively regulates autophagy, including inhibited autophagy protein ATG5, which implies that autophagy might be a downstream pathway of Hh signaling. Interestingly, both the Hh signaling pathway and autophagy are important regulators of osteoblastic differentiation and mineralization. Hh signaling was a positive regulator, whereas autophagy was a negative regulator signal on osteoblast differentiation. Most importantly, the Hh signaling pathway inducing osteoblast differentiation seems to occur by suppressing the autophagy pathway (Fig. 8). Our work reveals the mechanism underlying Hh signaling pathway regulation of bone development, and thus establishes a molecular basis for development of therapeutics for metabolic bone disorders.

## MATERIALS AND METHODS

### Fish handling and embryo preparation

Zebrafish (*Danio rerio*) wild-type AB strain was originally obtained from the College of Life Sciences and Technology, Tsinghua University. Fish feeding, breeding and maintenance were according to the literature (Kimmel et al., 1995). Embryos were obtained by natural mating; synchronous embryos at the appropriate stage were collected. Transgenic zebrafish lines Tg (-2.2col10a1a:GFP) were bought from CZRC (China Zebrafish Resource Center), fluorescence images were taken using a fluorescence microscope (Olympus, IX 51). This research was reviewed and approved by the Laboratory Animal Management and Animal Welfare Committee at the Institute of Medicinal Biotechnology of the Chinese Academy of Medical Sciences and all efforts were made to minimize the animals' suffering.

### Synthesis of MOs and microinjection

All morpholino oligos were synthesized and bought from Gene-tools LLC (http://www.gene-tools.com), 5MO and MisMO sequences were synthesized according to our previous published data (Hu et al., 2011; Hu et al., 2017). SHHMO sequence ′5-GCAGCACTCTCGTCAAAAGCCGCAT-3′ was according to Nasevicius and Ekker, (2000). Gli2MO/Ptch1MO was used to target the initiation site of *Gli2/Ptch1* mRNA and inhibit translation, Gli2Mis and Ptch1Mis was used as control, and designed by Gene Tools, LLC. The sequences were as follows. Gli2MO-5′ CTCCATGATGAGACTTCTTGGATGA3′; Gli2Mis:5′CTCgATcATcAGACTTgTTcGATGA3′; Ptch1MO-5′ACATTAACAGCCGAGGCCATGTTGC3′; Ptch1Mis-5′ACATaAAgAcCCGAGcCCATcTTGC3′. The 50 µM MOs were injected into 1–4 cell stage embryos.

### Drug treatment

10 mM stocking RAPA (provided by National Institutes for Food and Drug Control) solution in DMSO was added to the embryo media at final concentrations of 10 µM from 3 dpf to 6 dpf, and cyA (Sigma-Aldrich, C4116), were dissolved into DMSO, and then was diluted to 10 µM and 50 µM. 0.1% DMSO treated larvae was used as negative control. 3MA (Sigma-Aldrich, M9281) was dissolved into heated ddH_2_O.

### qPCR and RNA-seq

Larvae at 6 dpf were collected into Trizol Reagent (Sigma-Aldrich). Total RNA was extracted following the Trizol Reagent RNA extraction kit manual. First-strand cDNAs were synthesized by reverse transcription using the M-MLV RTase cDNA Synthesis Kit (Promega). qPCR primer pairs are listed in Table 1, parameters were 95°C 5 min, (95°C 15 s, 68°C 40 s), for 40 cycles. β-actin was amplified as a template loading control. Then the ct values were analyzed using a statistical calculation according to the instrument manual. WT group and 5MO-injected group larvae (30 larvae each group) at 6 dpf were sent to Compass biotechnology company for transcriptome sequencing, the mRNA-related Hh signaling and osteoblast were analyzed. RNA-seq data had been deposited with GEO under accession number GSE120170.

**Table 1.**
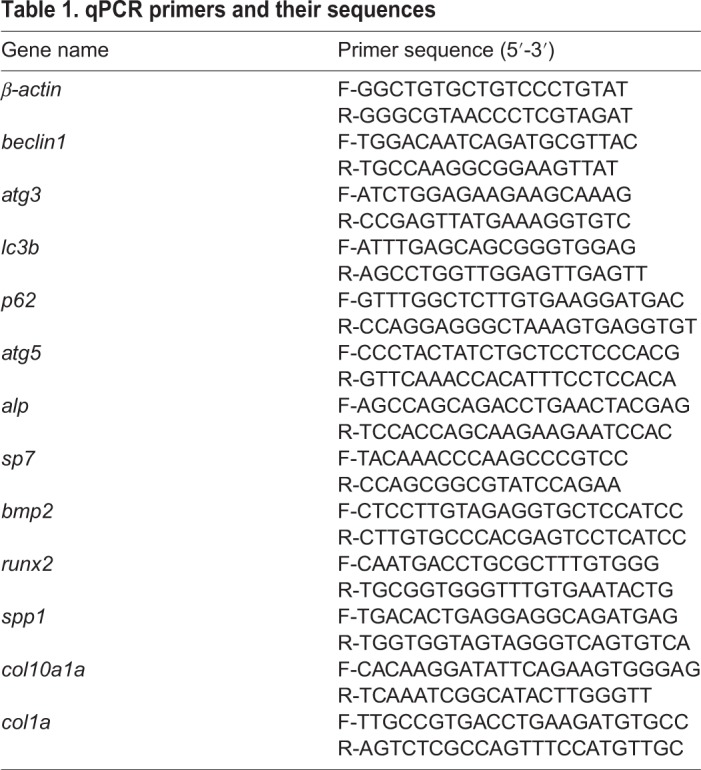
**qPCR primers and their sequences**

### Western blot

The procedure has been previously described in our previous literature (Hu et al., 2011). Briefly, zebrafish larvae total protein was extracted with the RIPA Lysis Kit (Applygen Technologies Inc, C1053), different protein samples were separated in 12% SDS-PAGE gel, then transferred to NC (nitrocellulose) membrane, and blocked with 5% milk in TBS for 40 min–1 h at room temperature. Then, the membranes were incubated with primary antibody at 4°C overnight, TBS washed three times and incubated with horseradish peroxidase-conjugated secondary antibodies 1 h at room temperature. The membranes were exposed to the Tanon 5200 Imaging System and we acquired the images. The primary antibody information is as follows: mouse anti-human β-GAPDH antibody (Zhongshan Goldbridge, TA08); anti-human ATG5 (Novus, NB110-53818), P62 rabbit polyclonal antibody (MBL, PM045), LC3B mouse monoclonal antibody (MBL, M186-3), SP7 (osterix) goat polyclonal antibody (Santa Cruz, sc-22538) and BMP2 Mouse Monoclonal antibody (Novus, MAB 1128).

### Alizarin Red staining

Larvae at 6 dpf were collected and fixed for 2 h by 4% paraformaldehyde at room temperature, fixed larvae were stained with 0.1% Alizarin Red (Sigma-Aldrich, A5533) in 0.5% KOH overnight. Stained larvae were gradient rehydrated with 75% ethanol (100 mM Tris pH 7.5, 10 mM MgCl_2_), 50% ethanol and 25% ethanol, bleached with 3% H_2_O_2_ containing 1% KOH for 20 min (Luo et al., 2016), and rinsed six times with ddH_2_O. Samples were then placed in 90% glycerol for imaging (Olympus, IX 51).

### Statistical analysis

Data in figures represent mean±s.d., derived from at least three experiments. Statistical analyses were performed using one-way ANOVA tests.
